# Can elderly individuals perform partial weight bearing on their lower limbs? A prospective cohort study using ambulatory real-time biofeedback

**DOI:** 10.1186/s13018-023-03807-4

**Published:** 2023-04-27

**Authors:** Tobias Peter Merkle, Nina Hofmann, Christian Knop, Tomas Da Silva

**Affiliations:** Department of Trauma Surgery and Orthopaedics, Klinikum Stuttgart – Katharinenhospital, Kriegsbergstraße 60, 70174 Stuttgart, Germany

**Keywords:** Partial weight bearing, Rehabilitation, Elderly, Orthosis, Biofeedback

## Abstract

**Background:**

Partial weight bearing in an orthosis and with forearm crutches is a widespread and well-accepted therapeutic principle after an injury of the lower extremity during early rehabilitation. Complying may be challenging to do under these circumstances, especially for elderly people. This study compares the spatiotemporal parameters and peak loads performed by a group of older participants before and after activating real-time biofeedback (BF) to determine whether they benefit from a biofeedback.

**Methods:**

Twenty-four healthy subjects between 61 and 80 years learned how to walk using forearm crutches in a lower leg orthosis while performing a weight of 20 kg using a bathroom scale with the aim of loading in a zone between 15 and 30 kg. After that, they completed a course that was on level ground (50 m) and another course on stairs (11 steps). They did a walk without BF first, and then with BF. Each step was given a maximum load, which was determined and statistically checked. In addition, spatiotemporal parameters were collected.

**Results:**

The classical teaching method with a bathroom scale was ineffective. Only 32.3% of the loads could be adequately carried by a person on level ground in the 15–30 kg target zone. On the stairs, it was 48.2% and 34.3%, respectively. Thus, on level ground, 52.7% of loads exceeded 30 kg. Downstairs it was 46.4%, and upstairs it was 41.6%. Subjects clearly benefit from activated biofeedback. Biofeedback significantly reduced missteps > 30 kg in every course. The loads decreased significantly to 25.0% on level ground, to 23.0% upstairs, and to 24.4% downstairs. At the same time, speed and stride length decreased per course while total time increased.

**Conclusion:**

Partial weight bearing is more complex and difficult for the elderly. These study results may help better understand 3-point gait in older adults in an outpatient setting. When partial weight bearing is recommended, special follow-up attention must be given for this group. Age-based therapy strategies can be developed and monitored with the assistance of ambulatory biofeedback devices.

*Trial registration* Retrospectively registered, https://www.drks.de/DRKS00031136.

## Background

A common orthopedic recommendation after surgery of the lower limb is to keep a partial load in an orthosis. Rehabilitation strategies aim to achieve a specific PWB with as little incorrect loads as possible. Overloading the extremity should be avoided in order to prevent osteosynthesis failure or mal union, and underloading on the contrary should be avoided in order to prevent thrombosis, nonunion, inactivity osteoporosis, or loss of strength and function [1, 2].

Preliminary research indicated that training using ambulatory biofeedback devices was superior to training with only bathroom scales or verbal instructions in terms of its effectiveness in assisting patients in adhering to partial weight-bearing instructions [3–6]. Such devices are increasingly being used in training to help patients comply with instructions to perform partial weight bearing [7–9] and have already been used successfully in younger people. However, complying PWB is particularly challenging for elderly people as walking is one of the most important activities in old age for maintaining an adequate quality of life [10, 11]. Particularly in old age, health is a prerequisite for independence and active participation in social life, making early postoperative mobilization essential. Elderly are particularly vulnerable to the negative outcomes that can result from bed rest and other forms of immobility. The patients’ quality of life and physical performance are further diminished by fear of falling again. Worrying about doing something wrong also plays role in therapy [12, 13].

The purpose of this study was to identify spatiotemporal parameters in a group of healthy elderly participants during partial load maintenance while wearing a lower leg-length orthosis (SP Air Smart Walker, Sporlastic, Nuertingen, Germany). In addition, we investigated whether or not older people were able to follow the instructions of a specific partial load and to what extent they benefited from switched-on biofeedback. This was accomplished by completing two courses: a 50-m walkway on the ground and a staircase with 11 steps.

## Materials and methods

### Participants

The study was approved by the medical ethics research committee of the University of Tübingen (protocol number 674/2021BO2). Before testing, a written consent form explaining the study was given to each subject. Twenty-four subjects (13 women and 11 men) with a minimum age of 60 years were recruited for this study. Only healthy subjects (ASA 1) according to ASA criteria were included in the study [14]. There were no orthopedic abnormalities or signs of cardiological, pulmonary, and neurological symptoms among the included participants, nor was there any history of cognitive disorders. Other subject characteristics were: weight 73.9 (57–100) kilogram, height 1.69 (1.48–1.85) meter, BMI 25.9 (22.0–29.8) kg/m^2^, age 70.0 (61–80) years, and shoe size 41 (38–46) EU.

### Instrumentation

**Fig. 1:**
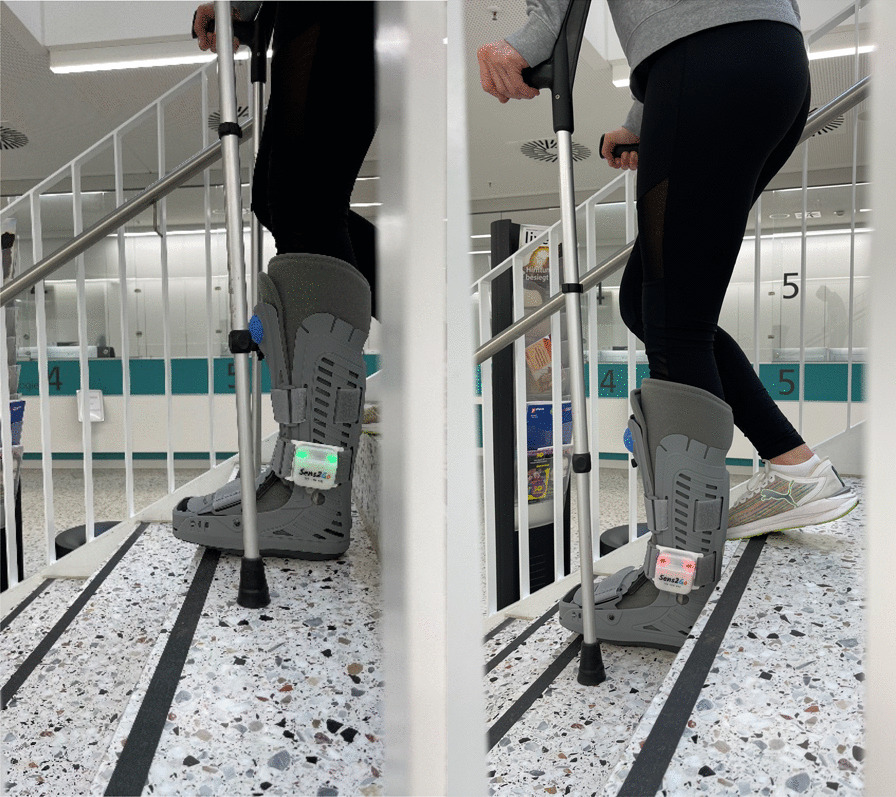
Loads under 20 kg results in a green light (left), over 20 kg in a red light accompanied by an audible “beep”-sound (right)

Our department's standard clinical procedure for treating ankle fractures was used as a template for adapting the study protocol. All study participants received a lower leg-length orthosis according to their foot size and crutches according to their height. The orthosis includes a built-in insole that measures the maximum load to each step. A device on the outside of the orthosis can be used to turn on or off audiovisual biofeedback for training purposes. Feedback is provided to the subjects in real-time. A green light displayed on the device if the weight was less than 20 kg. A red light showed that the patient was carrying too much load if the device detected a weight of more than 20 kg. Simultaneously with the red light, an auditory warning signal had been played. During the trial, no smart device was required. The data are wirelessly transferred in real-time to the examiner’s mobile phone and are saved in the app based for further analysis. The device is a CE-marked medical device (Sens2Go, Golex AG, Basel, Switzerland). The company declares that there is a 1% variability in the results of the measurement (Fig. 1).

Incompliance was defined as exceeding the 30 kg limit. The optimal load zone was determined to be the column weighing between 15 and 30 kg. In order to achieve this zone, participants learned to maintain a partial weight of 20 kg using a personal scale and walk with a three-point gait on flat ground and stairs. After confirming that they had learnt and understood the required partial load of 20 kg, the participants began two courses, first, without using biofeedback (the group serving as the control), and secondly using biofeedback (the group serving as the test arm). A 50-m walk on level ground (course 1) was followed by a run up and down the stairs (course 2). Each subject was permitted to run at their own pace. The examiner did not intervene throughout the examination to control the partial load. After completing the parkours without biofeedback, a pause was made to activate the biofeedback system. Additionally, the rest was taken to keep the subjects from becoming overextended. The examiner was only a passive observer and did not intervene in the examination with biofeedback. In order to avoid post-response learning, participants were not informed of their outcomes following their runs. These runs were performed twice, once with the left leg and once with the right, in order to determine the differences between the two sides. The data from both groups were compared with each other.

## Results

### Statistical analysis

Statistical analysis and graphing were performed using JASP (Computer software, Version 0.16.3, Department of Psychological Methods University of Amsterdam, Amsterdam, Netherlands). Data are reported as the mean and standard deviation. Significance was set at the level of *p* < 0.05.

### Spatiotemporal parameters

The group without biofeedback (control group) needed 173.9 (SD 81.0) seconds and 69.6 (SD 8.2) double steps to walk on level ground (50 m). The group with biofeedback (test group) needed 207.3 (SD 121.7) seconds and 78.2 (SD 25.2) double steps for the 50 m. These increases in the test group were not significant for both time (*p* = 0.116) and steps (*p* = 0.060).

The control group (no biofeedback) needed 44.6 (SD 21.3) seconds upstairs and 41.9 (SD 19.3) seconds downstairs. The test group (with biofeedback) needed 45.5 (SD 21.4) seconds upstairs (*p* = 0.841) and 43.9 (SD 19.6) seconds downstairs (*p* = 0.619) for the 11 steps. There were no significant differences between the left and right sides.

The following spatiotemporal parameters shown in Table 1 (course 1—level ground (50 m)) and Table 2 (course 2—stairs) were thereby calculated.

**Table 1:** Spatiotemporal parameters on level ground

Course 1—level ground (50 m)			
	(No biofeedback)	(With biofeedback)	*p*-value
Stride length (cm)	77.4 (SD 2.3)	70.9 (SD 2.4)	*p* = 0.179
Stride duration (s)	2.5 (SD 0.9)	2.6 (SD 1.2)	*p* = 0.578
Speed (m/s)	0.4 (SD 0.18)	0.3 (SD 0.190)	*p* = 0.423
Frequency (double steps/min)	27.1 (SD 8.5)	26.6 (SD 8.6)	*p* = 0.752

The mean and standard deviation for the crutch gait is presented. Significance level was set to 0.05

**Table 2:** Spatiotemporal parameters on stairs

Course 2—Stairs				
	Upstairs		Downstairs	
Stride duration (s)				
No BF	3.5 (SD 1.7)	*p* = 0.796	3.3 (SD 1.6)	*p* = 0.648
With BF	3.6 (SD 1.7)		3.4 (SD 1.5)	
Speed (staircase steps/s)				
No BF	0.30 (SD 0.3)	*p* = 0.824	0.31 (SD 0.117)	*p* = 0.483
With BF	0.29 (SD 0.3)		0.29 (SD 0.108)	
Frequency (double steps/min)				
No BF	20.4 (SD 7.1)	*p* = 0.814	22.1 (SD 9.3)	*p* = 0.311
With BF	19.1 (SD 7.5)		20.4 (SD 7.3)	

The mean and standard deviation for the crutch gait is presented. Significance level was set to 0.05

Activating biofeedback causes a not significant decrease in walking speed and a not significant increase in the total time required to complete a course. Accordingly, their stride duration is longer, and their frequency is lower. This is equally visible in both courses.

### Weight bearing

The number of maximum loads per step of all subjects is shown in Table 3. There were no significant differences between the left and right sides. Each step was assigned to a load column. 32.3% of the steps taken by the test group on level ground fell within the optimal load zone. It was 48.2% upstairs and 34.2% downstairs. Due to the biofeedback signal, the control group was able to take significantly fewer incorrect steps > 30 kg, resulting in a load shift to the lower load zones.

**Table 3:** Weight bearing of all users (runs *n* = 48)

	Biofeedback	< 15 kg (%)	15–30 kg (%)	> 30 kg (%)
*Course 1*				
Level ground 50 m	Without	15.0^a^	32.3^b^	52.7^c^
	With	29.7^a^	45.3^b^	25.0^c^
(a) *p* < 0.001; (b) *p* = 0.010; (c) *p* = 0.001				
Course 2				
Upstairs	Without	10.2^a^	48.2^b^	41.6^c^
	With	22.0^a^	55.0^b^	23.0^c^
(a) *p* = 0.005; (b) *p* = 0.319; (c) *p* = 0.012				
Downstairs	Without	19.3^a^	34.3^b^	46.4^c^
	With	33.8^a^	41.6^b^	24.4^c^
(a) *p* = 0.024; (b) *p* = 0.160; (c) *p* = 0.001				

The sum of the steps per load zone is given in %. Significance level was set to 0.05

None of the participants were able to maintain partial weight bearing for the entire distance. This was visible during both the run without and with the use of biofeedback. However, results showed that two groups of people loaded differently. One group were the efficient users, who could load at least 20% of all steps without overloading more than 20% > 30 kg. The other group consisted of high users, who exceeded 20% or more of all steps over 30 kg but were able to lower these incorrect steps by turning on biofeedback. While there were no significant differences in the three load groups among the efficient users, the high users were motivated to take fewer steps in the high load column and more steps in the lower load columns. These differences are presented in tabular form in Table 4 (efficient user) and Table 5 (high user).

**Table 4:** Weight bearing of the efficient users

Course	Biofeedback	< 15 kg (%)	15–30 kg (%)	> 30 kg (%)
Level ground 50 m	Without	34.4^a^	58.8^b^	6.8^c^
	With	34,8^a^	58.4^b^	6.8^c^
*p* = 0.737; (b) *p* = 0.867; (c) *p* = 0.955				
Upstairs	Without	20.5^a^	72.8^b^	6.7^c^
	With	32.4^a^	61.3^b^	6.3^c^
(a) *p* = 0.095; (b) *p* = 0.094; (c) *p* = 0.895				
Downstairs	Without	45.5^a^	47.0^b^	7.5^c^
	With	46.0^a^	46.2^b^	7.8^c^
(a) *p* = 0.712; (b) *p* = 0.870; (c) *p* = 0.868				

The sum of the steps per load zone is given in %. Significance level was set to 0.05. Level ground runs *n* = 12; upstairs runs *n* = 22; downstairs runs n = 14

**Table 5:** Weight bearing of the high users

Course	Biofeedback	< 15 kg (%)	15–30 kg (%)	> 30 kg (%)
Level ground 50 m	Without	8.5^a^	23.4^b^	68.1^c^
	With	28.0^a^	40.9^b^	31.1^c^
*p* < 0.001; (b) *p* = 0.002; (c) *p* < 0.001				
Upstairs	Without	1.5^a^	27.5^b^	71.0^c^
	With	13.1^a^	49.7^b^	37.2^c^
(a) *p* = 0.002; (b) *p* = 0.003; (c) *p* < 0.001				
Downstairs	Without	8.5^a^	29.1^b^	62.4^c^
	With	28.9^a^	39.9^b^	31.2^c^
(a) *p* < 0.001; (b) *p* = 0.064; (c) *p* < 0.001				

The sum of the steps per load zone is given in %. Significance level was set to 0.05. Level ground runs *n* = 36; upstairs runs *n* = 26; downstairs runs *n* = 34

## Discussion

This is the first study to objectively evaluate the efficacy of an insole orthosis with a specified partial load in a sample of healthy older participants.

### Spatiotemporal parameters

Few studies have examined spatiotemporal metrics during partial weight bearing with crutches, and accordingly little is known about how their use affects gait parameters, particularly in the elderly [11]. In the present study, participants using biofeedback had a higher strait duration, while strait length, speed, and frequency decreased compared to the non-biofeedback group in all two courses. Samson characterized this as a natural consequence of aging [10]. A collection of mobility parameters has not been addressed in prior research involving ambulatory biofeedback devices. However, it must be noted that these systems have not yet been configured to collect such parameters by their manufacturers. The spatiotemporal data that were collected for this study are primarily descriptive and can be used as a basis for additional studies. These basis values may be used in in a clinical setting to determine if an individual is impaired relative to healthy subjects of the same age and gender. Understanding how biofeedback training may be used is an important step in developing rehabilitation strategies to help improve individual outcomes to reduce the risk of over- and underuse.

Several authors demonstrated in their studies that the gait of older persons who need a walking aid is more irregular and unstable than the gait of older adults who are independently mobile which consequently results in more energy expenditure [11, 15]. Older people need special attention. In view of the increase in these patient groups, early rehabilitation must also adapt to this group of patients [16]. We assume that elderly patients are prone to an inadequate implementation of the physiotherapists’ instructions. Therefore, real-time biofeedback devices can be a suitable tool for training in this group [17, 18].

### Partial weight bearing

This study's findings confirmed the results of other studies that had found that biofeedback is superior to traditional scale therapy for achieving a specific partial load [3–5]. Participants benefited from using a real-time biofeedback system. Incorrect loads > 30 kg could be significantly reduced with biofeedback in both courses. However, even with real-time biofeedback enabled, none of the participants were able to avoid undesired loads greater than 30 kg over the entire distance. Without BF, approximately two-thirds of the steps on level ground and downstairs were not loaded within the target zone. By activating the BF, the number of steps within the target zone increased to 45.3% and 41.6%, respectively. Upstairs, the percentage increased from 48.2 to 55.0%. Thus, biofeedback therapy is an approach to motivate a user to remain within a certain range.

### Limitations and strengths of the study

Only healthy people were looked at in this study. Subjects with a history of musculoskeletal, neurological, cardiovascular, or pulmonary diseases, as well as those who were underweight (BMI < 18.5) or overweight (BMI > 30.0), were excluded. It must be considered that patients will have substantially more difficulty maintaining a defined partial load, especially if more comorbidities can be assumed [4, 19, 20]. Nevertheless, we believe its important to study healthy individual’s movement sequence and pattern. These findings may help researchers better understand gait in patients and distinguish between gait abnormalities caused by the use of a walking assistance.

We are aware that medical–technical services are only one component of therapeutic assistance designed to compensating for specific deficiencies that impede physical functioning. These include important senses, which are often the first to decline with age [21]. Vision compensates for balance imbalances initially. Those with hearing impairments find it more difficult to follow verbal instructions or audio biofeedback, particularly in a noisy environment. The use of multiple forms of biofeedback, such as audio, visual, and haptic biofeedback, at the same time is especially important in old age. The orthosis itself transmits an audiovisual biofeedback. The mobile phone, which must be worn close to the body, can generate a haptic signal additionally. Because all subjects were able to perceive it sufficiently, only a purely audiovisual BF was used in this study. All individuals rated the system’s live feedback positively, and they reported a subjective increase in their sense of security.

According to the findings of this study, the biofeedback system used in this study is effective in elderly people, which suggests that it may have significant clinical relevance among partially weight-bearing orthopedic patients in higher age. Clinical trials with patients must be conducted to determine whether patients also could implement this. There remains a significant need for research in this field.

## Conclusion

Independence and security are important factors to consider in old age. Furthermore, keeping mobility in old age is essential for the health of joints, bone, and muscles. A fracture of the lower limbs severely limits elderly patients' mobility in particular. Modern real-time biofeedback systems can help improve safety and comfort. The system utilized in this study has the potential to be used in the future to support elderly individuals in preserving their independence following accidents and to create therapeutic concepts. Their full potential, however, requires additional testing in patient populations, particularly the elderly.

## Data Availability

The datasets during and/or analyzed during the current study available from the corresponding author on reasonable request.

## Abbreviations

BF: Biofeedback
PWB: Partial weight bearing
